# Supplementary material for: PLGA Based Nanospheres as a Potent Macrophage-Specific Drug Delivery System
Source: Nanomaterials (Basel). 2021 Mar 16;11(3):749. doi: 10.3390/nano11030749 (PMC8002218; doi:10.3390/nano11030749)
Supplement: Supplementary file 1 [file nanomaterials-11-00749-s001.pdf]

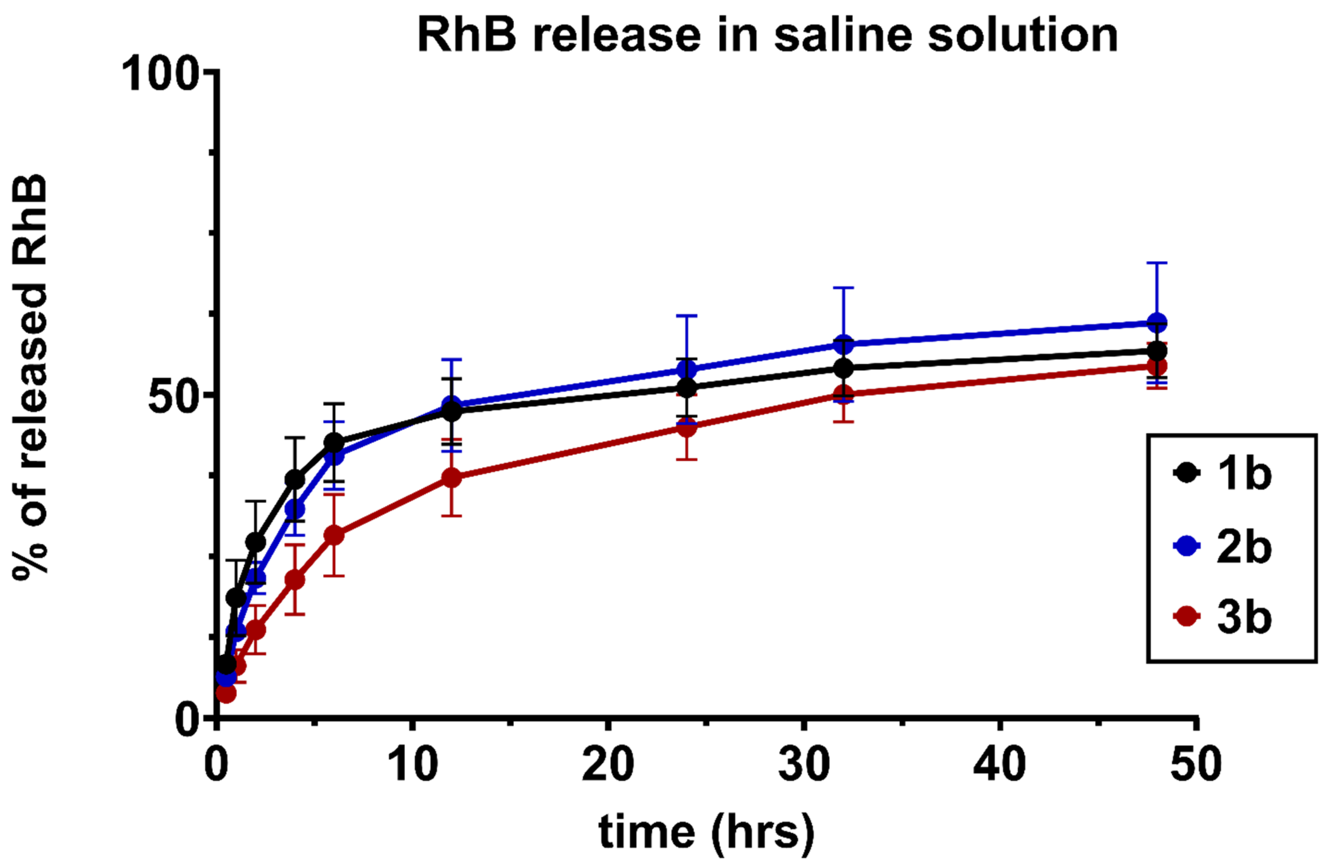

RhB release from PLGA nanospheres study was performed using dialysis technique. Briefly, 1ml of purified nanosuspension was added into dialysis bag (molecular weight cut-off: 5 500 Da). The dialysis bag was emersed into 10 ml of PBS and put to the shaking water bath that mas kept 37°C. Samples (10 ml) were taken at different time intervals and the whole volume of the sample was replaced with fresh PBS. The amount of released RhB was evaluated using UV-VIS spectrophotometry at wavelength 555 nm.
